# 2067. Preclinical Characterization of Rationally Designed Microbial Consortia to Prevent Bacterial Infections in Patients with Chronic Liver Disease

**DOI:** 10.1093/ofid/ofad500.137

**Published:** 2023-11-27

**Authors:** Elizabeth Halvorsen, Marin Vulić, Kelsey Barrasso, Nicholas Beauchemin, Jessica Brown, Nathaniel J Ennis, Melissa Mayol, Jenna Wurster, Edward J O’Brien, Christopher Ford, Matthew Henn

**Affiliations:** Seres Therapeutics, Cambridge, MA; Seres Therapeutics, Cambridge, MA; Seres Therapeutics, Cambridge, MA; Seres Therapeutics, Cambridge, MA; Seres Therapeutics, Cambridge, MA; Seres Therapeutics, Cambridge, MA; Seres Therapeutics, Cambridge, MA; Seres Therapeutics, Cambridge, MA; Seres Therapeutics, Cambridge, MA; Seres Therapeutics, Inc, Cambridge, MA; Seres Therapeutics, Cambridge, MA, Cambridge, Massachusetts

## Abstract

**Background:**

In chronic liver disease (CLD), gut microbiome disruption worsens with disease severity and growing evidence suggests it is a key driver of infection risk through a combination of intestinal pathogen expansion from a loss of colonization resistance and dysfunction of the gastrointestinal (GI) barrier. For the SER-147 preclinical program, we evaluated rationally designed cultivated consortia of human commensal bacteria in vitro and in vivo for the ability to reduce the abundance of pathogens commonly observed in patients with CLD, such as *Enterococcus* and *Enterobacteriaceae*.

**Methods:**

Candidate consortia for SER-147 were designed by leveraging genomic data from public observational CLD patient studies and Seres interventional human trial data to include taxa associated with reduced risk of infection and select strains from Seres’ strain library to maximize features associated with decolonizing key pathogens. We developed a novel *in vitro* gut-ecology model (iGEM) system that propagates human stool-derived microbial communities to evaluate candidate consortia for their ability to reduce titers of vancomycin-resistant *Enterococcus faecium* (VRE), and both carbapenem-resistant *Klebsiella pneumoniae* (CR-Kpn) and *Escherichia coli* (CR-Ec). *In vivo*, candidate consortia were evaluated for their ability to reduce fecal titers of VRE and CR-Kpn in mouse models of gut pathogen colonization.

**Results:**

In the iGEM system, following introduction and expansion of select pathogens in the microbial community, intervention with a subset of five unique candidate consortia from the SER-147 program were able to significantly reduce titers of VRE and Cr-Ec compared to controls and in the case of Cr-Kpn, titers were reduced to the limit of detection of the assay (Figure 1). In the *in vivo* colonization models, oral administration of the same five candidate consortia led to a 1-3 Log_10_ reduction in VRE and Cr-Kpn fecal titers compared to vehicle-only treated mice (Figure 2).

**Figure 1.**
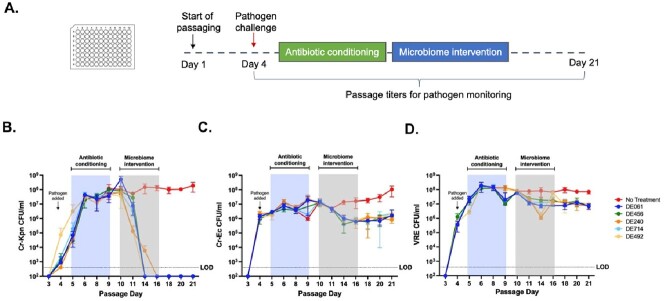
Reduction in abundance of Cr-Kpn, Cr-Ec, and VRE after microbiome intervention in the iGEM system.

(A) Human stool-derived microbial communities were passaged anaerobically in 96-well deep well plates, with cultures stamped into fresh deep well plates every 24 hours with different wells representing different treatments and conditions. Individual pathogens were introduced on day 4 of the experiment, followed by a vancomycin pre-treatment from days 5-8. On days 10-16, five unique candidate consortia (DE061, DE456, DE240, DE714, and DE492) were dosed daily as the microbiome intervention. (B, C, D) The titers of Cr-Kpn, Cr-Ec, or VRE were quantified at specific timepoints during the study by plating culture aliquots on selective and differential media. The mean CFU per milliliter of culture was calculated for each arm of the study and plotted on the line graph (N=4 per arm). Error bars represent the standard deviation from the mean. L.O.D., limit of detection.

**Figure 2:**
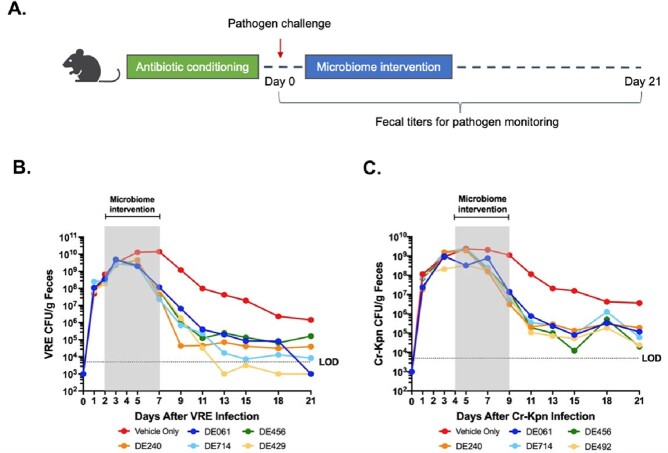
Reduction in abundance of Cr-Kpn and VRE after microbiome intervention in mouse models of gut pathogen colonization.

(A) Wild-type C57BL/6 mice underwent antibiotic conditioning prior to pathogen challenge. Following the introduction of pathogen (Day 0) mice were dosed daily vial oral gavage with five unique candidate consortia (DE061, DE456, DE240, DE714, and DE492) as the microbiome intervention or vehicle only for 6 days and fecal titers are monitored for a 3-week period to evaluate pathogen abundance (B,C) The titers of VRE or CR-Kpn were quantified in fecal pellets by plating on selective agar at the indicated time-points. The median (B) CR-Kpn and (C) VRE CFU per gram of feces was calculated for each group and plotted on the line graph (n=9 per group). L.O.D., limit of detection.

**Conclusion:**

Preclinical assessments *in vitro* and *in vivo* support the ability of SER-147 candidate consortia to reduce VRE, CR-Kpn, and CR-Ec abundance in the gut. These results support the potential ability of microbiome therapeutics to reduce the risk of infection in patients with CLD.

**Disclosures:**

**Elizabeth Halvorsen, PhD**, Seres Therapeutics: Employee|Seres Therapeutics: Stocks/Bonds **Marin Vulić, PhD**, Seres Therapeutics: Employee|Seres Therapeutics: Stocks/Bonds **Kelsey Barrasso, PhD**, Seres Therapeutics: Stocks/Bonds **Nicholas Beauchemin, n/a**, Seres Therapeutics: Employee|Seres Therapeutics: Stocks/Bonds **Jessica Brown, n/a**, Seres Therapeutics: Employee|Seres Therapeutics: Stocks/Bonds **Nathaniel J. Ennis, n/a**, Seres Therapeutics: Employee|Seres Therapeutics: Stocks/Bonds **Melissa Mayol, n/a**, Seres Therapeutics: Employee|Seres Therapeutics: Stocks/Bonds **Jenna Wurster, PhD**, Seres Therapeutics: Paid Employee|Seres Therapeutics: Stocks/Bonds **Edward J. O'Brien, PhD**, Seres Therapeutics: Employee|Seres Therapeutics: Stocks/Bonds **Christopher Ford, PhD**, Seres Therapeutics: Employee|Seres Therapeutics: Stocks/Bonds **Matthew Henn, PhD**, Seres Therapeutics: Employee|Seres Therapeutics: Stocks/Bonds

